# Bactofencin A Displays a Delayed Killing Effect on a Clinical Strain of *Staphylococcus aureus* Which Is Greatly Accelerated in the Presence of Nisin

**DOI:** 10.3390/antibiotics14020184

**Published:** 2025-02-11

**Authors:** Paula M. O’Connor, Paul D. Cotter, Colin Hill, R. Paul Ross

**Affiliations:** 1 Teagasc Food Research Centre, Moorepark, Co. Cork, P61 C996 Fermoy, Ireland; paula.oconnor@teagasc.ie (P.M.O.); paul.cotter@teagasc.ie (P.D.C.); 2 APC Microbiome Ireland, University College Cork, T12 YT20 Cork, Ireland; c.hill@ucc.ie; 3 School of Microbiology, University College Cork, T12 YT20 Cork, Ireland

**Keywords:** bactofencin A, nisin A, synergy, mode of action, mastitis, *Staphylococcus aureus*

## Abstract

**Background/objectives:** Bacteriocins can be considered a novel source of natural alternatives to antibiotics or chemical food additives with the potential to fight against clinical and food pathogens. A number have already been commercialised as food preservatives, but they also have the potential to treat drug-resistant clinical pathogens and can play a role in immune modulation. To achieve their full potential, an understanding of their mode of action is required. **Methods:** Bactofencin A and nisin A were purified to homogeneity by reversed-phase HPLC and their effect on the mastitis pathogen *Staphylococcus aureus* DPC5246 was assessed by cell viability assays and flow cytometry. **Results:** We report that bactofencin A displays a delayed inhibitory effect against the mastitis pathogen, *Staphylococcus aureus* DPC5246, suggesting an unusual mode of action. This characteristic was clearly visible on BHI plate media, where formation of inhibition zones against the staphylococcal strain took 23 h compared to 6 h for the well-characterised nisin. This delayed killing and injury was also demonstrated using flow cytometry, where damage was evident 4 h after bacteriocin addition. Treatment with 2 μM bactofencin A resulted in approximately 20-fold higher numbers of injured and 50-fold higher numbers of dead cells when compared to untreated cells. Combining bactofencin A with the lantibiotic nisin A resulted in faster killing at lower bacteriocin concentrations. When combined in an equal ratio, the combination exhibited a 4-fold increase in inhibition compared to nisin A alone. These results demonstrate that the combination may be very effective in therapeutic applications against pathogenic staphylococci.

## 1. Introduction

Antimicrobial resistance (AMR) is a worsening global public health crisis that has been associated with overuse and misuse of antibiotics in both human and animal welfare cases. The emergence of multidrug-resistant pathogens threatens to undo a century of medical advances, placing anticancer treatments and routine surgeries at risk. At present, at least 700,000 people die worldwide each year from superbugs that arise due to AMR, and it has been predicted that this could lead to 10 million deaths by 2050 unless action is taken [1,2]. Indeed, in 2019, methicillin-resistant *Staphylococcus aureus* (MRSA) alone resulted in greater than 100,000 AMR-attributable deaths, and since then, the percentage of MRSA has increased from 1.84 in 2019 to 2.81 in 2022 in clinical isolates from patients in Italy [3,4]. It is also expected that health expenditure will have to increase by USD 300 billion to USD 1 trillion per year to deal with this problem, as patients are sick for longer and require more healthcare interventions and more expensive drugs to combat their illness [5,6]. The cost of AMR to the global economy is also significant and, when combined with healthcare costs, could rise to USD 60–USD 100 trillion per year by 2050 [6,7,8]. In 2017, the WHO published a report highlighting the lack of new antimicrobials in development against priority pathogens and the need for new classes of antimicrobials to combat the crisis [9]. More recently, the WHO launched an AMR Action Fund in collaboration with financial investors and the pharmaceutical industry to ensure a sustainable pipeline of new antibiotics effective against superbugs, with a specific aim of developing two to four new antimicrobial treatments for patients by 2030 [10]. Alternative therapeutic avenues using antibodies, probiotics, vaccines, and antimicrobial peptides are now attracting increasing attention in the fight against AMR [11].

Bacteriocins are ribosomally synthesised antimicrobial peptides, produced by most genera of bacteria, that can have a broad or narrow spectra of inhibition [12]. The production of bacteriocins by Generally Regarded as Safe (GRAS) strains makes them of particular interest to the food industry where they (mainly nisin) have been used as food biopreservatives [13]. The increase in AMR has meant that new antimicrobials are required, which is why bacteriocins are being considered as an alternative for certain applications. In this respect, their stability, low toxicity, target specificity, and activity against a broad range of bacteria, including antibiotic-resistant pathogens, present distinct advantages [14]. They are ribosomally synthesised and can be bioengineered, an approach that has been effective for nisin in producing variants with improved activity against certain pathogens [15]. Antimicrobial efficacy can also be improved through combining bacteriocins, particularly those with different modes of action [16]. In addition, recent studies suggest that commensal bacteriocin producers are modulators of the human microbiome with potential to play a role in treating intestinal infections [17,18].

Bactofencin A, produced by the porcine gut isolate *Lactobacillus salivarius* DPC6502, is a Class IId bacteriocin that is particularly potent against *Staphylococcus aureus*. Immunity is thought to be due to the action of a DltB homologue that may increase dealanylation of teichoic acids, thereby reducing the negative charge on the cell wall and preventing binding of the cationic bactofencin A. Bactofencin A is a twenty-two amino acid bacteriocin with a positively charged N terminal containing a series of positively charged amino acids (KRKKHR) and a C terminal loop formed via a disulphide bond between Cys7 and Cys22 [19]. Charge and structure play a significant role in its potency and its proposed mode of action is through an initial attraction to the cell membrane via the cationic N terminal, with inhibition occurring through interaction of the C terminal loop with a putative receptor. Bactofencin A has also been shown to affect subtle changes in the microbiome with the potential to inhibit anaerobic inhabitants such as *Clostridium* and *Bacteroides* [20].

Nisin A is a Class I lantibiotic produced by some strains of *Lactococcus lactis* that displays broad-spectrum activity against most Gram-positive microorganisms [21]. It has been used as a food preservative by the food industry since the 1950s [12] and, more recently, its use has been extended to biomedical applications, including inhibition of drug-resistant pathogens such as MRSA, enterococci and *Clostridioides difficile* [22]. Nisin A is a thirty-four amino acid peptide containing five lanthionine rings [23] that are responsible for its intrinsic stability and potent activity, the latter of which is often reported at nanomolar concentrations [24,25]. Nisin exerts its antimicrobial effect by way of multiple modes of action, including blocking of cell wall biosynthesis through lipid II binding, pore formation and, most recently, DNA condensation [26,27,28]. It is noteworthy that significant spontaneous nisin resistance rarely occurs in nature, despite widespread use in the food industry and this is attributed to its multiple modes of action [22].

Combining bacteriocins with other antimicrobials, referred to as antimicrobial combinatorial therapy, has the dual advantage of improving potency while reducing the incidence of AMR. Furthermore, these combinations can result in cheaper treatments and reduced toxicity to the host due to the lower antimicrobial concentrations required for effective treatment [29]. Recently, this approach has been used to improve potential treatments against the foodborne clinical pathogen, *S. aureus*. Specifically, the lantibiotic nisin has shown promising results when assessed in combination with other antimicrobial compounds, including citric acid [30], essential oils [31], antibiotics [32,33,34], phage endolysins [35], fruit extract [36], and other bacteriocins [37].

The aim of this study was to assess the effectiveness of bactofencin A and nisin A, both alone and in combination, at killing the mastitis isolate *S. aureus* DPC5246.

## 2. Results

Bactofencin A is particularly effective against *S. aureus*, including MRSA strains [19]. In this study, we tested the effectiveness of bactofencin A alone and combined with nisin A against a strain that was isolated from the milk of a cow with mastitis (inflammation of the udder). The delayed inhibition displayed by bactofencin A and its inability to fully lyse the target culture required an alternative approach to the traditional 96-well assay procedures for assessment of inhibition, minimal inhibitory concentration (MIC), and fractional inhibitory concentration (FIC) measurements. Consequently, MIC assays were carried out at 4 mL scale to provide a sufficient volume for cell number enumeration at intervals throughout the growth time period.

### 2.1. The Inhibitory Effect of Increasing Concentrations of Bactofencin A on S. aureus DPC5246

**Figure 1:**
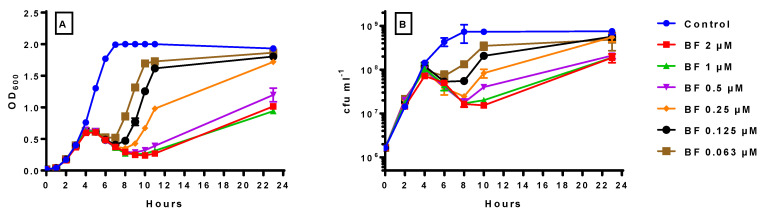
Inhibitory effect of 0.063, 0.125, 0.25, 0.50, 1 and 2 μM bactofencin A on *S. aureus* DPC5246 in BHI broth at 37 °C as measured by OD_600_ (**A**) and viable cell counts (cfu mL^−1^) (**B**). Growth experiments were carried out in duplicate and standard deviation displayed as error bars on the graphs.

Initially, the effect of a wide range of 2-fold increasing concentrations of bactofencin A (0.063, 0.125, 0.250, 0.5, 1 and 2 µM) on the OD_600_ and viable cell numbers of a growing culture of *S. aureus* DPC5246 was measured. The optical density readings (Figure 1A) suggest that bactofencin A had little or no effect on growth for the first 3–4 h regardless of concentration. After 4 h, the growth rate of bactofencin A-treated cultures slowed compared to the control; this became more apparent from 5 h onwards, when the OD_600_ decreased, suggesting cell lysis. After 7 h, the cultures appeared to recover in a concentration-dependent manner, with samples containing lower bactofencin A concentrations recovering before those containing higher concentrations. Interestingly, after 23 h, the OD_600_ for higher bactofencin A concentrations (0.5, 1 and 2 μM) remained lower than the control, suggesting that bactofencin A was still having an inhibitory effect on the culture at these concentrations.

The viable count results (Figure 1B) showed a similar delayed response with no significant difference in viability after 2 h, while at 4 h, a 0.4-log reduction was detected in cell numbers for 2 μM bactofencin A. At 6 h, all bactofencin A concentrations showed a 1-log reduction compared to the control, with the exception of 0.063 μM, which had a slightly lower impact (0.7 log), while at 8 h, the maximum effect was achieved, with bactofencin A concentrations between 0.5 and 2 µM showing a 1.5-log reduction compared to the control. Taken together, the OD_600_ and viable plate count results suggest a delayed action of the bacteriocin on the culture that eventually leads to significant cell lysis and subsequent regrowth.

### 2.2. Flow Cytometry Analysis of S. aureus DPC5246 Grown in the Presence of 0, 0.2, and 2 μM Bactofencin A

Flow cytometry can be used to study the real-time effect of bacteriocins on cell membranes through the use of fluorescent dyes such as thiazole orange (TO) and propidium iodide (PI) [38]. TO can enter live cells, whereas PI can only label cells with compromised membranes, thereby allowing distinction between live (green), injured (orange), and dead (red) cells. The separation of each cell type through gating optimisation results in a reliable assay that can quantify the number of each cell type. Here, we used flow cytometry to study the effect of bactofencin A on *S. aureus* DPC5246 cells.

**Figure 2:**
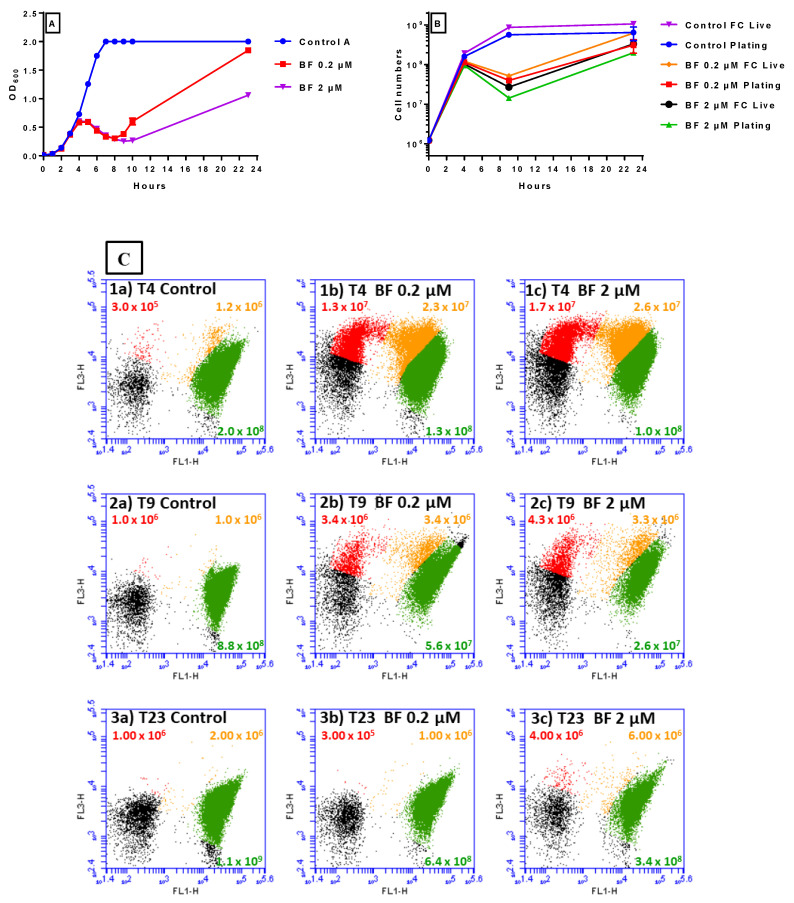
(**A**,**B**) The effect of 0 (control), 0.2 and 2 μM bactofencin A on cell viability of *S. aureus* DPC5246 at 4, 9, and 23 h, as measured by OD_600_ (**A**); cell numbers determined by flow cytometry (FC Live) and conventional plating (plating) (**B**). Growth experiments were carried out in duplicate and the standard deviation is displayed as the error bars shown in the graphs. (**C**) The effect of 0 (control), 0.2 and 2 μM bactofencin A on cell viability of *S. aureus* DPC5246 at 4, 9, and 23 h, as measured by flow cytometry. (**C**) (**1a**,**1b**,**1c**) are 0 (control) μM bactofencin A, 0.2 and 2 μM bactofencin A at 4 h, respectively, (**2a**,**2b**,**2c**) are 0 (control) μM bactofencin A, 0.2 and 2 μM bactofencin A at 9 h, respectively, and (**3a**,**3b**,**3c**) are 0 (control) μM bactofencin A, 0.2 and 2 μM bactofncn A at 23 h, respectively.

*S. aureus* DPC5246 was grown in BHI broth in the presence of 0, 0.2 and 2 μM bactofencin A and growth assessed hourly by OD_600_ (Figure 2A). The OD_600_ results showed that 0.2 and 2 μM bactofencin A inhibited the culture compared to the untreated control, resulting in reductions in OD_600_ following a dose–response behaviour.

Comparison of cell numbers by traditional plating and flow cytometry at 4, 9, and 23 h compared very well (Figure 2B). The OD_600_ and cell enumeration results confirm that bactofencin A has minimal effect on growth in the first 4 h of exposure with maximum killing occurring at 8 h, followed by a limited recovery of the culture at 23 h for both concentrations tested.

In addition to enumerating live cells (green), the flow cytometry results, which were corrected to exclude background debris (black), show the number of dead (red) and injured (orange) cells (Figure 2C) at 4, 9 and 23 h. At 4 h, the untreated control contains 2 × 10^8^ live cells and about 100-fold fewer injured cells (1.2 × 10^6^) and dead cells (3 × 10^6^) which is indicative of a “healthy” log phase culture (Figure 2C(1a)). In comparison, the 0.2 μM (Figure 2C(1b)) and 2 μM bactofencin A (Figure 2C(1c))-treated cultures contained slightly fewer live cells than the untreated control (1.3 × 10^8^ and 1.0 × 10^8^) but approximately 20-fold higher levels of injured (2.3 × 10^7^ and 2.6 × 10^7^) and 50-fold higher numbers of dead cells (1.3 × 10^7^ and 1.7 × 10^7^). At 9 h, the effect of bactofencin A on live cells was apparent, as the untreated control sample contained 8.8 × 10^8^ (Figure 2C(2a)) compared to a 1.3–1.6-log reduction (5.6 × 10^7^ and 2.6 × 10^7^) detected for 0.2 μM (Figure 2C(2b)) and 2 μM (Figure 2C(2c)) bactofencin A, respectively. However, the number of injured (3.4 × 10^6^, and 3.3 × 10^6^) and dead cells (3.4 × 10^6^ and 4.3 × 10^6^) was now just 3-fold higher than the control sample (1.0 × 10^6^). At 23 h, we again observed that bactofencin A had a slight inhibitory effect on the culture as the untreated control contained 1.1 × 10^9^ live cells (Figure 2C(3a)) while 0.2 μM bactofencin A (Figure 2C(3b)) contained 6.4 × 10^8^ and 0.2 μM bactofencin A (Figure 2C(3c)) contained 3.4 × 10^8^ live cells. Notably, at this stage, the numbers of injured and dead cells are comparable to the untreated control.

In summary, flow cytometry suggests that bactofencin A causes considerable damage to the cell within 4 h of exposure, suggesting that the antimicrobial effect is delayed.

### 2.3. Antimicrobial Interaction Between Bactofencin A and Nisin A on an S. aureus DPC5246 Indicator Plate

**Figure 3:**
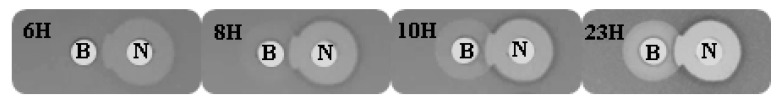
Antimicrobial interaction between 10 μM bactofencin A (B) and 10 μM nisin A (N) against *S. aureus* DPC5246 at 6, 8, 10, and 23 h.

Inhibition of *S. aureus* DPC5246 by bactofencin A and nisin A was assessed by well diffusion assay. The indicator plate (Figure 3) showed that the zone of inhibition for bactofencin A appeared gradually between 8 and 23 h, reflecting the growth curve results (Figure 1A) and again confirming a delayed action by the bacteriocin. In contrast, the zone of inhibition for nisin A was already apparent within 6 h, confirming the rapid killing effect of nisin. Interestingly, the nisin zone was bigger in the region where the two bacteriocin zones intersected, suggesting the possibility of a positive interaction between the two antimicrobials.

### 2.4. Inhibitory Effect of Bactofencin A and Nisin A Alone on Growth of S. aureus DPC5246

**Figure 4:**
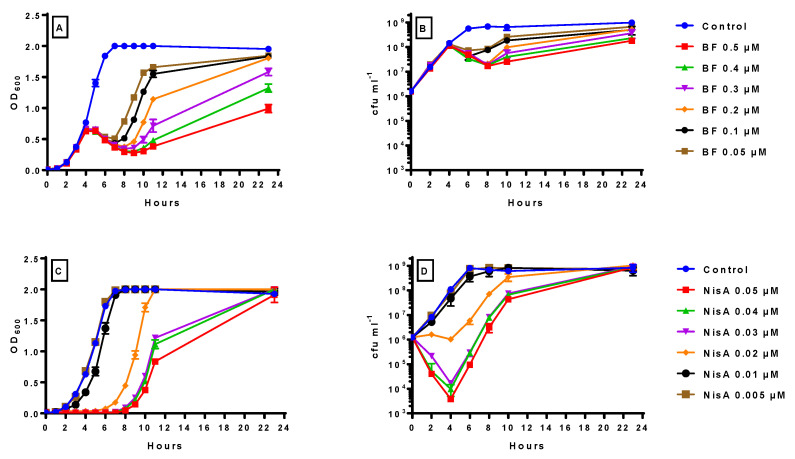
Inhibitory effect of 0.05–0.5 μM bactofencin A on OD_600_ (**A**) and cfu mL^−1^ (**B**) and 0.005–0.05 μM nisin A on OD_600_ (**C**) and cfu mL^−1^ (**D**) of *S. aureus* DPC5246 in BHI broth at 37 °C. Growth experiments were carried out in duplicate and the standard deviation is displayed as error bars shown in the graphs.

The observation that bactofencin activity is enhanced by nisin prompted us to investigate whether this phenomenon could be observed in liquid media. The effect of a narrow range of concentrations of bactofencin A (between 0.05 and 0.5 µM) and nisin A at 10-fold lower concentrations (0.005 to 0.05 µM) on *S. aureus* DPC5246 was assessed with a view to selecting optimum concentrations for combinatorial studies. The OD_600_ results (Figure 4A) for the bactofencin A concentration range again showed a delayed inhibition curve for all bactofencin concentrations tested in line with the trends seen over a wider bactofencin A concentration range in Figure 1A and Figure 2A. The plate count results (Figure 4B) showed no significant reduction in viable cell counts compared to the control at 4 h and optimum killing at 8 h, once again confirming a delayed killing effect.

Nisin A was assayed at a 10-fold lower concentration range (0.05–0.005 µM) than bactofencin A, given its greater potency at earlier stages of growth. One thing to note is that higher nisin A concentrations inhibit the cultures immediately (Figure 4C) and also that the delay in recovery is concentration-dependent. At 23 h, all nisin A-treated cultures grew to the same extent as the untreated control, regardless of the nisin A concentration. The plate count results (Figure 4D) show a similar trend in terms of viability, where the higher concentrations reduced the viability of the culture for the first 4 h, which then recovered in a dose-dependent fashion.

Given that nisin and bactofencin seemed to act in concert in the plate assay, we then evaluated the effect of different bactofencin A/nisin A combinations.

### 2.5. Inhibitory Effect of Bactofencin A/Nisin A Combinations

**Figure 5:**
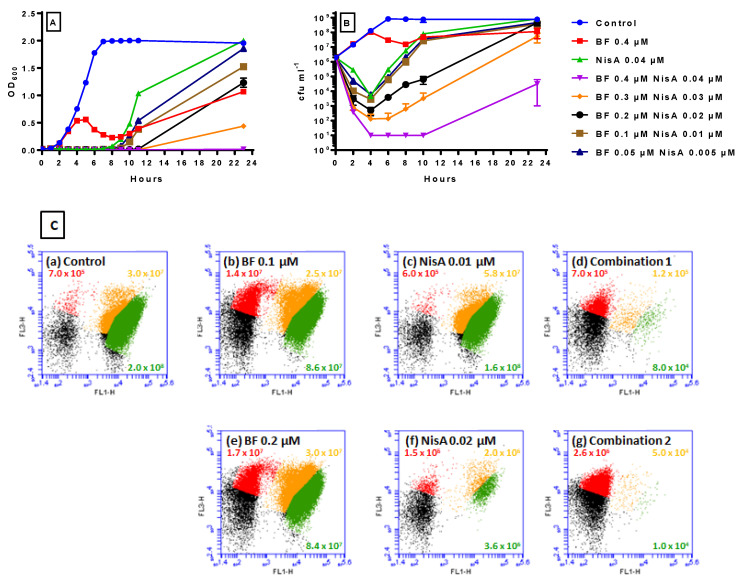
The effect of bactofencin A/nisin A combinations on *S. aureus* DPC5246 as measured by OD_600_ (**A**) and cfu mL^−1^ (**B**) and flow cytometry of bactofencin A 0.1 or 0.2 µM alone, nisin A 0.01 or 0.02 µM alone, and bactofencin A/nisin A 0.1/0.01 or 0.2/0.02 µM combinations at 4 h (**C**). Growth experiments were carried out in duplicate and standard deviation is displayed as the error bars shown in the graphs. (**C**) The effect of bactofencin A/nisin A combinations on *S. aureus* DPC5246 as measured by flow cytometry at 4 h. (**a**) 0 µM control, (**b**) 0.1 µM bactofencin A, (**c**) 0.1 µM nisin A, (**d**) 0.01 µM bactofencin A/nisin A, (**e**) 0.2 µM bactofencin A, (**f**) 0.1 µM nisin A, and (**g**) 0.01 µM bactofencin A/nisin A.

Initially, bactofencin A (0.05–0.5 µM) was combined with nisin A (0.05–0.005 µM) to assess their effect on growth of *S. aureus* DPC5246 (Figure 5). Bactofencin A 0.4 µM and nisin A 0.04 µM were included as controls.

The optical density results (Figure 5A) showed that the untreated control was fully grown at 6 h; 0.4 µM bactofencin A showed delayed inhibition, while 0.04 µM nisin inhibited the culture for 7 h, after which it recovered to levels comparable to those of the untreated control. Interestingly, all bactofencin A/nisin A combinations suppressed growth for 8 h. The two lowest combinations, bactofencin A 0.05 µM/nisin A 0.005 µM and bactofencin A 0.1 µM/nisin A 0.01 µM, inhibited *S. aureus* DPC5246 for 9 h (Figure 5). This is noteworthy, considering that nisin A alone at these concentrations has no significant effect on growth (see Figure 4C), while equivalent bactofencin A concentrations are already recovering (Figure 4A), suggesting possible additive or synergistic action between the two bacteriocins. At 23 h, the inhibitory effects of the bacteriocin combinations are dose-dependent, with the OD_600_ for the highest combination tested being comparable to the OD_600_ at T0, suggesting complete suppression of culture growth.

The plate count results (Figure 5B) confirmed that the delayed killing effect of bactofencin A no longer occurs in the presence of all concentrations of nisin. The lower combinations are equivalent to nisin A 0.04 µM alone, while the higher combinations are significantly better, with the highest combination resulting in complete killing of the culture. Overall, we see that both killing and regrowth occur in a dose–response manner.

Flow cytometry was used to assess the effect of the lower bactofencin A/nisin A combinations on live, dead, and injured cell numbers at 4 h. The untreated control at 4 h (Figure 5C(a)) was similar to that shown in Figure 2C with 2 × 10^8^ live cells, and 3.0 × 10^7^ injured cells, again commensurate with a control untreated culture. The numbers of live, dead, and injured cells are comparable for bactofencin A alone at 0.1 (Figure 5C(b)) and 0.2 (Figure 5C(e)) µM, and it is interesting to note the high proportion of injured cells even at the lower bactofencin A concentration. The number of live and injured cells in the presence of nisin A 0.01 µM alone was similar to the control, though more injured cells were detected (5.8 × 10^7^ vs. 3.0 × 10^7^), while nisin 0.02 µM resulted in an almost 2-log reduction in live cells compared to the control, in agreement with Figure 4D. Notably, both combinations resulted in a 3–4-log reduction in cell numbers, demonstrating the effectiveness of the combinations compared to either bacteriocin alone.

### 2.6. The Effect of Decreasing Nisin A Concentrations in Combination with 0.4 µM Bactofencin A and Decreasing Bactofencin A Concentrations in Combination with 0.04 µM Nisin A on S. aureus DPC5246

**Figure 6:**
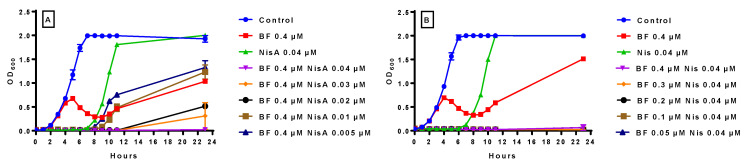
Inhibitory effect of decreasing concentrations of nisin A (0.04–0.005 μM) in the presence of 0.4 μM bactofencin A on OD_600_ (**A**) and decreasing concentrations of bactofencin A (0.4–0.05 μM) in the presence of 0.04 μM nisin A on OD_600_ (**B**) on growth of *S. aureus* DPC5246 in BHI broth at 37 °C. Growth experiments were carried out in duplicate and standard deviation is displayed as the error bars shown in the graphs.

The effect of decreasing nisin A concentrations in relation to bactofencin A (Figure 6A) and decreasing bactofencin A in relation to nisin A (Figure 6B) was assessed to determine the contribution each bacteriocin makes to activity. In both experiments (Figure 6A,B), the control samples (untreated control, bactofencin A alone and nisin A alone) were similar to those shown in Figure 5A. Treatment of *S. aureus* DPC5462 with 0.04 µM bactofencin A and decreasing nisin A concentrations resulted in inhibition of culture for 7 h at all combinations tested (Figure 6A). Recovery occurred in a dose–response manner, as evidenced by bactofencin A 0.4 µM/nisin A 0.005 µM and bactofencin A 0.4 µM/nisin A 0.01 µM starting to recover at 8 and 9 h, respectively, while the three higher nisin A concentrations were still inhibitory at 11 h. At 23 h, there was a reduction in OD_600_ at all bactofencin A/nisin A combinations assayed compared to the untreated control, while bactofencin A 0.4 µM/nisin A 0.04 µM was totally inhibited. Overall, the results suggest that while the cultures are significantly inhibited at all combinations tested, decreasing the nisin A concentration results in less inhibition.

In contrast to the constant bactofencin A and decreasing nisin A concentrations, the OD_600_ does not recover in samples with constant nisin A 0.04 µM and decreasing bactofencin A concentrations (Figure 6B), as inhibition is maintained for 23 h, suggesting that bactofencin A levels can be reduced once sufficient nisin A is present.

### 2.7. Fractional Inhibition of S. aureus DPC5246 with Bactofencin A and Nisin A on a S. aureus DPC5246 Indicator Plate

**Figure 7:**
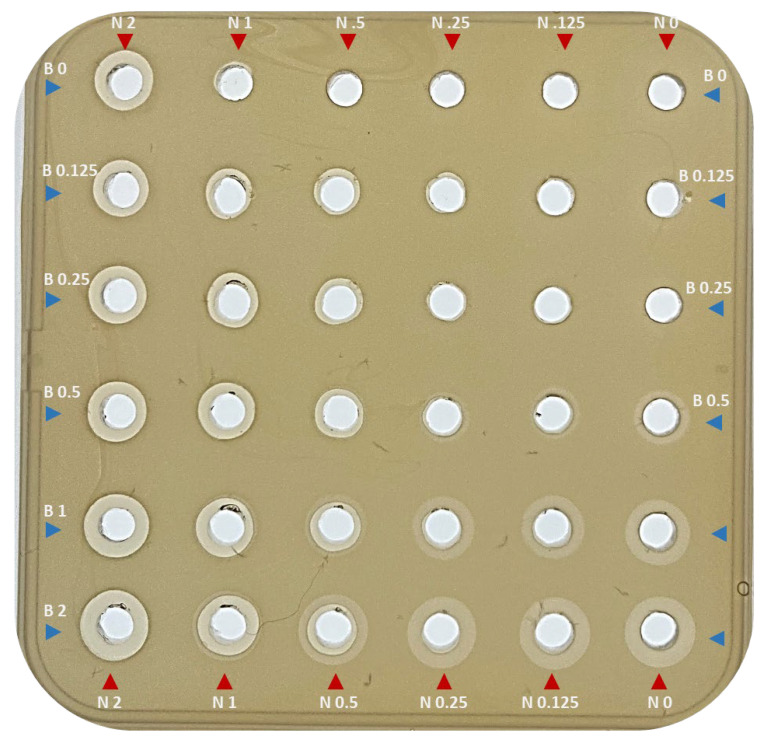
Inhibitory effect of decreasing concentrations of nisin A (2, 1, 0.5, 0.25, and 0.125 μM, horizontally across the plate) in combination with increasing concentrations of bactofencin A (2, 1, 0.5, 0.25, and 0.125 μM, vertically down the plate) on *S. aureus* DPC5246.

Finally, a fractional inhibition-type assay was used to assess the effect of 2×, 1×, 0.5×, 0.25× and 0.125× combinations of nisin A and bactofencin A on *S. aureus* DC5246 by agar well diffusion, where 1 µM of each peptide is considered 1×. The indicator plate (Figure 7) shows that nisin A alone results in a zone of clearing at 2 µM but not at 1, 0.5, 0.25, or 0.125 µM, while bactofencin A alone results in hazy zones at 2, 1, and 0.5 µM.

The addition of low concentrations (0.125 or 0.25 µM) of nisin A does not enhance the bactofencin A activity against *S. aureus* DPC5246, as no difference in zone size or appearance is observed at any bactofencin A concentration. In contrast, the addition of 0.125 µM bactofencin A, the lowest concentration tested, results in a clear zone of inhibition in the presence of 0.5 µM and 1 µM nisin A, again showing that bactofencin A levels can be reduced once sufficient nisin A is present.

Another notable feature is the clear zone surrounded by a hazy zone for 0.5 µM nisin A when assayed with 1 µM and 2 µM bactofencin A, providing further evidence that bactofencin A concentrations can be reduced once sufficient nisin A is present.

## 3. Discussion

Bactofencin A is a novel bacteriocin with potential to fight infection as, in addition to its potency against *S. aureus*, its small size and lack of post-translational modifications make it amenable to peptide synthesis. Synthetic bactofencin A is as active as the naturally produced peptide, and the disulphide bonds form naturally over time, making it a suitable source of peptide for characterisation studies [39]. Interestingly, the studies presented here demonstrate that the bacteriocin has a delayed action when compared to nisin and almost certainly has a very different mechanism. Initially, stock solutions of bactofencin A and nisin A were assessed for peptide purity by reversed-phase HPLC and MALDI TOF mass spectrometry and the presence of a single HPLC peak containing the correct peptide mass was taken as evidence of sufficient purity for inhibition studies (Supplementary Figure S1).

Class II bacteriocins typically act through an initial electrostatic interaction with negatively charged components of the cell membrane, and in some cases (class IIa in particular) have been shown to bind to a cell receptor, resulting in a loss of ion gradients, membrane integrity, and cell death [40,41]. It has been tentatively proposed that bactofencin A interacts with the cell wall via the positively charged N terminal and also binds to an unknown receptor by interaction with the C terminal half of the peptide, given that some of these are essential for activity [39].

The delayed action of bactofencin A on *S. aureus* DPC5246 (Figure 1), in addition to the cell damage observed at 4 h by flow cytometry (Figure 2) and the absence of significant cell lysis, suggest that bactofencin A executes its antimicrobial action via a mechanism that takes considerable time, in some cases up to several generations, to result in cell death. Cell disruption by bactofencin A may simply be due to the strong interaction between the bacteriocin and the cell surface due to its strong positive charge (+7 at neutral pH) and an as-yet-unknown receptor; further research is required to identify this possible receptor. The ability of 10-fold lower levels of nisin A (Figure 4) to completely inhibit the culture may be attributable to the different modes of action as pore-forming bacteriocins, such as nisin, often act at nanomolar concentrations while cell wall disrupters require higher peptide concentrations to exert an effect [42]. Combining bactofencin A with 10-fold less nisin A resulted in increased killing compared to either bacteriocin alone (Figure 5), again suggesting that the two different modes of action are complementary to each other. Lowering the bactofencin A concentration in relation to nisin A resulted in greater inhibition compared to lowering the nisin A concentration in relation to bactofencin A (Figure 6 and Figure 7), suggesting that bactofencin A makes *S. aureus* more susceptible to inhibition by nisin A (Figure 7).

Nisin is a pentacyclic peptide consisting of N terminal rings, A, B, and C, linked to the C terminal rings, D and E, via a three-amino-acid flexible hinge region [23]. The N terminal rings are proposed to form a pyrophosphate cage around lipid II, thereby preventing cell wall synthesis. A stable pore is then formed when the C terminal rings, facilitated by the flexible hinge, insert across the membrane [43,44,45]. These pores are proposed to increase in size through nisin–lipid II aggregation, resulting in catastrophic damage to the cell [44,46]. It is possible that the changes resulting from exposure to bactofencin A could enhance nisin A–lipid II interactions, thereby increasing the effectiveness of nisin A.

Nisin immunity in the producing strain is mediated through NisI, a dedicated immunity protein that intercepts nisin, preventing it from binding to Lipid II, and an ABC transporter, nisFEG, that ejects nisin from the cell membrane [44,47,48]. Nisin resistance mechanisms have been found in non-lantibiotic-producing strains and include changes in target specificity, cell wall and cell membrane modifications, specific nisin degradation enzymes, and two-component systems that resemble the LanFEG immunity systems found in lantibiotic gene clusters [49,50].

D-alanylation of teichoic acids via DltA is a specific nisin resistance mechanism seen in *S. aureus*. Teichoic acids, including lipoteichoic acids attached to the cell membrane (LTA) and wall teichoic acids (WTA) attached to peptidoglycan, are major components of Gram-positive cell walls that play a role in adhesion, growth, virulence, and biofilm formation [51,52,53]. The addition of D-alanine esters to teichoic acids via the D-alanyl lipoteichoic acid (DLT) pathway reduces the overall negative charge on the cell wall, making the cell more resistant to cationic peptides [52]. In *S. aureus*, the DLT pathway proteins are encoded on the *dlt* operon, *dltABCD*, with DltA catalysing the alanylation of D-alanine in the cytoplasm and transferring it to DltC, a D-alanyl carrier protein [52,53]. Activated DltC forms a tight complex with DltB, a channel-/funnel-forming acyltransferase that moves the activated D-alanine across the membrane where DltD, which is located outside the cell membrane, transfers it to the teichoic acids [54]. Interestingly, the DLT pathway, and DltB in particular, have been proposed as targets for drug-resistant *S. aureus* infections [55]. Indeed, numerous Gram-positive bacteria with mutated *dlt* genes have a higher negative charge on the cell wall, making them more susceptible to cationic AMPs [56]. Furthermore, wild-type *S. aureus* strains with extra copies of the *dlt* operon result in teichoic acid with increased D alanylation, making the cell more positively charged and, consequently, more resistant to cationic antimicrobial peptides [57].

*S. aureus* contains 16 two-/three-component regulatory systems (TCSs) that sense and respond to environmental changes [58,59]. These include the BraRS (Bacitracin resistance-associated Response System), GraRS (Gramicidin/Glycopeptide resistance-associated Response System) and VraRS (Vancomycin resistance-associated Response System), and TCSs that confer natural resistance to low levels of nisin in *S. aureus* via sensor kinases and response regulators [49,60]. Specifically, the sensor kinase BraS, GraS, or VraS detects environmental stress such as nisin and the regulators BraR, GraR, and VraR activate genes that help to reduce binding of AMPs. This resistance is typically mediated through expression of BraDE/VraDE, ABC transporters that confer nisin resistance and also through activation of the *dlt* operon described above [61]. Interestingly, single-point mutations in BraS or BraR result in constitutive expression of VraDe, making *S. aureus* tolerant to high concentrations of nisin A [61], while inactivation of any of these three TCS results in increased sensitivity to nisin A [62,63,64]. NsaRS (Nisin stress-associated Response System) is another TCS that confers nisin resistance similar to BraRS, GraRS, and VraRS in *S. aureus* [65]. This response system protects the cell through the regulation of the ABC transporter NsaAB when nisin is detected [65]. Overall, these TCs regulate the expression of genes that protect the cell envelope, allowing the cell to adapt and survive.

It is tempting to suggest that bactofencin A could enhance nisin A activity through targeting one of the nisin resistance mechanisms in *S. aureus*. The delayed action of bactofencin A suggests that it disrupts cell wall synthesis. As immunity to bactofencin A is conferred through a Dlt analogue that reduces the charge on the cell wall, bactofencin A could theoretically bind to teichoic acids via hydrogen bonding, resulting in changes to the cell that would allow it to act synergistically with nisin A. Teichoic acids interact with and regulate the activity of the autolytic enzymes, N-acetylmuranomoyl-L-alanine amidase and N acetylglucosaminidase, ensuring excessive peptidoglycan hydrolysis does not occur during cell growth and division. Interestingly, nisin A and pep 5 can induce autolysis in *S. aureus* by binding to teichoic acid and lipoteichoic acid, leading to uncontrolled cleavage of peptidoglycan [66].

Although this research focuses on a single strain of *S. aureus*, the synergy between bactofencin A and nisin A is likely to extend to other staphylococcal strains, including MRSA strains. Interestingly, Jensen et al. (2020) [28] report similar MIC values for nisin A against MRSA and non-MRSA strains, suggesting that methicillin resistance has no effect on nisin susceptibility. Evaluating more staphylococcal strains and, indeed, pathogens from different genera will be the subject of further investigation. Given the broad spectrum of inhibition of nisin activity, observations are likely to extend to other bactofencin-sensitive strains.

The increased efficacy of bactofencin A/nisin A combinations against the mastitis strain *S. aureus* DPC5246 is expected to have a number of positive benefits, in line with other antimicrobial combinatorial therapies. Specifically, reducing bacteriocin levels results in more cost-effective treatments, together with reduced toxicity potential to the host and reduced incidence of AMR. To develop the therapeutic potential of using bactofencin A/nisin A combinations as a treatment for mastitis, clinical studies are required to determine the efficacy of the antimicrobials at the site of infection and to assess host tolerance/toxicity, particularly for bactofencin A, as its toxicity levels are as yet unknown.

In summary, bactofencin A is very effective against *S. aureus* when combined with nisin A, a phenomenon that is most likely due to them acting synergistically through two different modes of action. While the mode of action of nisin A is well characterised, further work is required to determine the exact mode of action of bactofencin A.

## 4. Materials and Methods

### 4.1. Staphylococcus aureus DPC5246

*S. aureus* DPC5246 [67] is a bovine mastitis clinical strain isolated from a cow at Teagasc, Moorepark, Fermoy, Co Cork, which was selected for this study due to its association with mastitis. It was grown aerobically in BHI broth at 37 °C.

### 4.2. Bactofencin A Synthesis and Purification

Bactofencin A, KRKKHRCRVYNNGMPTGMYRWC, was synthesised using microwave-assisted solid-phase peptide synthesis (MW-SPPS) on a Liberty Blue microwave peptide synthesizer (CEM Corporation. Mathews, NC, USA) and purified by reversed-phase HPLC according to the method described by O’Connor et al. (2018) [39].

### 4.3. Purification of Nisin A

Nisin A was purified from nisinA^®^P provided by Handary SA (Brussels, Belgium) by reversed-phase HPLC. Specifically, 60 mg of nisinA^®^P was resuspended at 10 mg mL^−1^ in Milli Q water and 2 mL aliquots run on a semi-preparative, Jupiter Proteo (10 × 250 mm, 4 µ, 90 Å), reversed-phase HPLC column (Phenomenex, Cheshire, UK) running a 25–45% acetonitrile gradient, over 40 min, where mobile phase A was 0.1% trifluoroacetic acid (TFA) and mobile phase B was 100% acetonitrile 0.1% TFA. Eluent was monitored at 214 nm and fractions collected at 30 s intervals. Fractions containing nisin A were assayed to confirm the molecular mass of nisin A (3352 Da) by MALDI TOF mass spectrometry and those deemed pure were pooled and lyophilised.

### 4.4. Preparation of Peptides for Activity Assays

Bactofencin A was resuspended in 50 mM sodium phosphate buffer pH 6.8 at 1000 µM, while nisin A was resuspended in Milli Q water at 1000 µM. Resuspended peptides were assessed for purity before their use by analytical reversed-phase HPLC. Next, 15 µL of bactofencin A was added to 135 µL of Milli Q water and a 100 µL aliquot run on an analytical Aeris Peptide (4.6 × 250 mm, 5µ, 100 Å) reversed-phase HPLC column (Phenomenex, Cheshire, UK) running a 10–30% gradient over 40 min, where mobile phase A was 0.1% TFA and mobile phase B was 100% acetonitrile 0.1% TFA. Eluent was monitored at 214 nm and fractions were collected at approximately 1 min intervals. Bactofencin A eluted as a single peak and the bactofencin A-containing fraction was assessed for the bactofencin A mass (2782 Da) by MALDI TOF mass spectrometry (Supplementary Figure S1A).

Nisin A (3352 Da) was assessed as described for bactofencin A, although a 20–50% acetonitrile 0.1% TFA gradient was used (Supplementary Figure S1B).

### 4.5. MALDI TOF Mass Spectrometry

MALDI TOF mass spectrometry was performed on HPLC fractions of interest from purification protocols and resuspended pure peptides using an Axima TOF^2^ MALDI TOF mass spectrometer (Shimadzu Biotech, Manchester, UK). A 0.5 µL aliquot of matrix solution (α-cyano 4-hydroxy cinnamic acid, 10 mg/mL in 50% acetonitrile—0.1% TFA) was deposited onto the target and left for 20 s before being removed. The residual solution was allowed to air-dry and 0.5 µL sample solution was deposited onto the pre-coated sample spot. Then, 0.5 µL of matrix solution was added to the deposited sample and allowed to air-dry. The sample was subsequently analysed in positive-ion linear or reflectron mode.

### 4.6. Effect of Bactofencin A on the Growth of S. aureus DPC5246

An overnight culture of *S. aureus* DPC5246 was diluted 200-fold in BHI broth, resulting in a 0.5% inoculum containing ~1 × 10^6^ colony-forming units/mL (cfu mL^−1^). A 100 μM stock solution of bactofencin A was 2-fold serially diluted in 100 μL aliquots of BHI broth and 80 μL aliquots of each dilution were added to 3920 μL of the 0.5% inoculum, resulting in 4 mL samples containing 2, 1, 0.5, 0.25, 0.125, and 0.063 μM bactofencin A. Four millilitres of the inoculum without bactofencin A was included as a control. Samples were prepared in duplicate and incubated in a 37 °C water bath and their growth was measured spectrophotometrically (Jenway 6300 spectrophotometer, Cole-Parmer Jenway, Vernon Hills, IL, USA) via optical density at 600 nm (OD_600_) at hourly intervals. Viable cells were enumerated by determining cfu mL^−1^ at 0, 2, 4, 6, 8, 10, and 23 h. Specifically, 100 μL aliquots of sample were 10-fold serially diluted in 900 µL Maximum Recovery Diluent (MRD) and 10 µL aliquots of each dilution were spotted onto BHI agar plates and allowed to dry. Plates were incubated overnight at 37 °C and the cfu mL^−1^ was calculated at each time point.

### 4.7. Data Analysis

Growth experiments were carried out in duplicate and the data points are the average of two values. Error bars are displayed on the graphs.

### 4.8. Assessment of Cell Viability Via Flow Cytometry

The proportion of live, injured, and dead cells in *S. aureus* DPC5246 cultures grown in the presence of bactofencin A, nisin A and bactofencin A/nisin A combinations was assessed by flow cytometry at 4, 9, and 23 h. Cells were stained with a BD^TM^ Cell Viability Kit, which uses thiazole orange (TO) and propidium iodide (PI) to distinguish live and dead cell populations. Cultures for assay were diluted to ~ 10^6^ cells mL^−1^ in a staining buffer, which was phosphate-buffered saline containing 0.01% Tween 80 and 1 mmol/L EDTA. Next, 2 µL of each dye (TO and PI) was added to 200 µL of diluted sample and analysed on a BD Accuri^TM^ C6 flow cytometer (Becton, Dickinson and Company, BD Biosciences, San Jose, CA, USA). Gates, used to distinguish between live and dead cells, were assigned using the BD Accuri’s C6 associated software version 1.0.264.21 in line with the manufacturer’s guidelines.

### 4.9. Inhibition of S. aureus DPC5246 by Bactofencin A and Nisin A Assayed by Agar Well Diffusion

Inhibition of *S. aureus* DPC5246 by bactofencin A and nisin A was demonstrated initially by the agar well diffusion assay described by Ryan et al. (1996) [68]. An indicator plate containing *S. aureus* DPC5246 was prepared by adding 225 μL of an overnight culture to 45 mL of molten BHI agar (0.5% inoculum) which was allowed to solidify in a 120 mm square Petri dish. Then, 50 µL aliquots of 10 µM bactofencin A were placed close to 50 µL of 10 µM nisin A in pre-bored wells and the plate incubated at 37 °C. The plate was photographed at 6, 8, 10, and 23 h.

### 4.10. Inhibition Studies with Bactofencin A and Nisin A

Next, 4 ml samples were prepared by adding aliquots of 10 μM bactofencin A and/or 10 µM nisin A to the *S. aureus* DPC5246 inoculum containing ~ 1 × 10^6^ cfu mL^−1^ to provide the required bacteriocin concentration. Four millilitres of the inoculum without bactofencin A or nisin A was included as a control. Samples were prepared in duplicate and incubated in a 37 °C water bath. OD_600_ was recorded every hour. Viable cells were enumerated, where required, by determining cfu mL^−1^ at 0, 2, 4, 6, 8, 10, and 23 h, as described above.

### 4.11. Fractional Inhibition of S. aureus DPC5246 with Bactofencin A and Nisin A

Fractional inhibition of *S. aureus* DPC5246 with bactofencin A and nisin A was assessed by agar well diffusion assay. Finally, 4 μM stock solutions of bactofencin A and nisin A were individually 2-fold serially diluted in 300 µL BHI broth to create 4, 2, 1, 0.5, and 0.25 μM stock solutions. A checkerboard-type assay was set up by adding 25 μL aliquots of each bactofencin A concentration to 25 μL of each nisin A concentration in pre-bored wells. The agar plate was incubated at 37 °C and photographed at 23 h.

## Data Availability

The original contributions presented in this study are included in the article/Supplementary Materials. Further inquiries can be directed to the corresponding author.

## Supplementary Materials

The following supporting information can be downloaded at https://www.mdpi.com/article/10.3390/antibiotics14020184/s1, Figure S1: Assessment of peptide purity of bactofencin A (A) and nisin A; (B) stock solutions by Reversed Phase HPLC and MALDI TOF mass spectrometry.
