# Supplementary material for: Bactofencin A Displays a Delayed Killing Effect on a Clinical Strain of Staphylococcus aureus Which Is Greatly Accelerated in the Presence of Nisin
Source: Antibiotics (Basel). 2025 Feb 11;14(2):184. doi: 10.3390/antibiotics14020184 (PMC11851555; doi:10.3390/antibiotics14020184)

Supplementary Figure S1

Purification of peptides for activity assays

The stock solutions of bactofencin A and nisin A were assessed for purity prior to use in activity assays by analytical HPLC and MALDI TOF Mass Spectrometry.

Bactofencin A eluted as a single peak at 29 minutes on the HPLC chromatogram and MALDI TOF mass spectrometry of the bactofencin A containing fraction detected a mass of 2782 Da (Supplementary Figure S1A) which is within the expected mass range (2782 +/- 1 Da) for bactofencin A. Nisin A also eluted as a single peak at 31 minutes and MALDI TOF mass spectrometry detected a mass of 3353 Da in the nisin A containing fraction which is also within the expected range (3352 Da +/- 1 Da) (Supplementary Figure S1B).

Supplementary Figure S1 Assessment of peptide purity of bactofencin A (A) and nisin A (B) stock solutions by Reversed Phase HPLC and MALDI TOF mass spectrometry.

(Ai) Bactofencin A

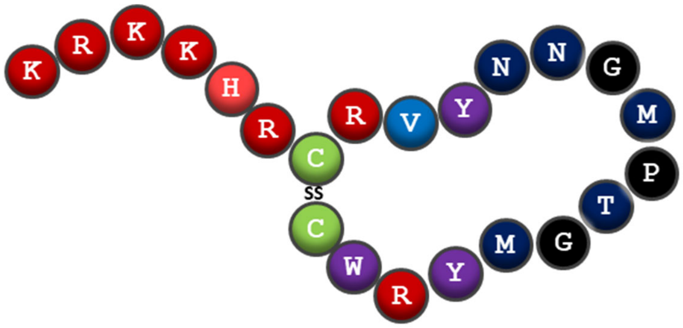

(Aii) Bactofencin A HPLC and MALDI TOF MS

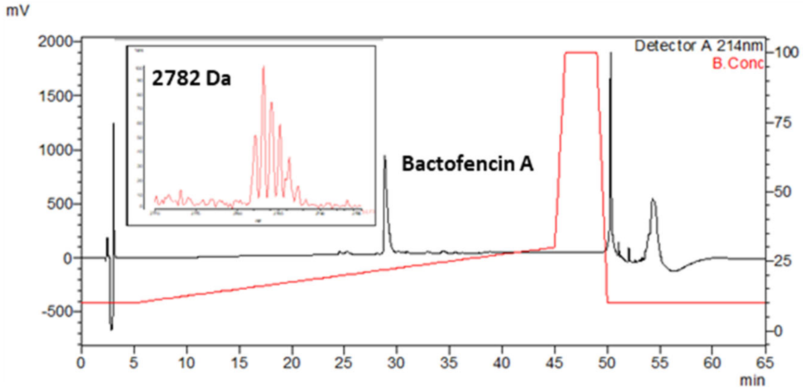

(Bi) Nisin A

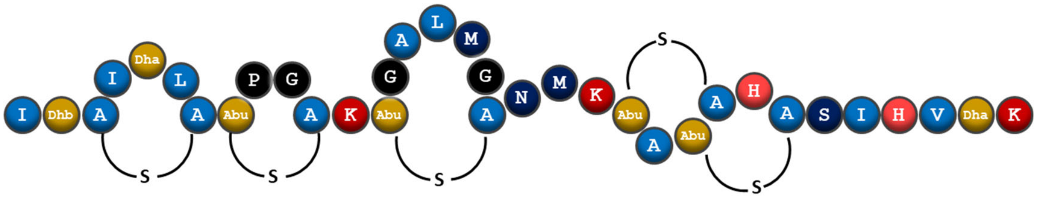

(Bii) Nisin A HPLC and MALDI TOF MS

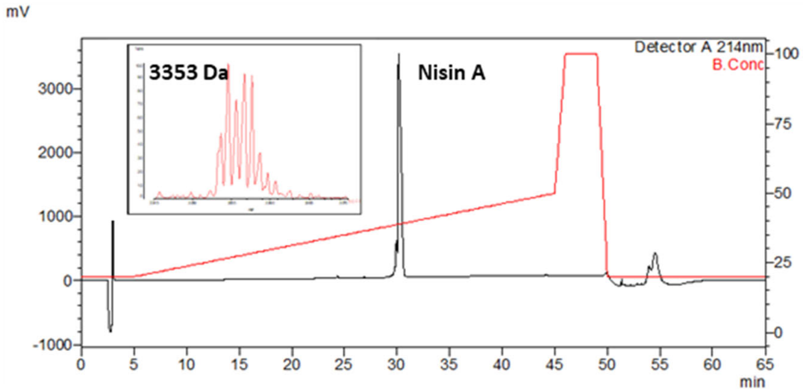

Supplement: Supplementary file 1 [file antibiotics-14-00184-s001.zip › antibiotics-3436028-supplementary.pdf]
